# Hepatic, Periportal, Retroperitoneal, and Mesenteric Neurofibromatosis in von Recklinghausen's Disease

**DOI:** 10.7759/cureus.2248

**Published:** 2018-02-28

**Authors:** Kumail Khandwala, Zafar Sajjad, Summar-un-nisa Abbasi, Muhammad Usman Tariq

**Affiliations:** 1 Department of Radiology, The Aga Khan University, Karachi.; 2 Department of Radiology, The Aga Khan University Hospital, Karachi.; 3 Department of Pathology & Laboratory Medicine, The Aga Khan University, Karachi.

**Keywords:** plexiform neurofibroma, neurofibromatosis, hepatic hilum, retroperitoneum, mesentery

## Abstract

We present a rare case of histologically proven neurofibromatosis of the liver, hepatic hilum, retroperitoneum, and mesentery. An adult male who had been diagnosed with neurofibromatosis (NF) type 1 underwent a computed tomography (CT) scan for abdominal pain and vomiting. The CT scan showed a large low-attenuating lesion in the region of porta hepatis which was infiltrating along portal tracts into the liver, encasing the major vessels, and extending into the retroperitoneum and mesentery. Based on the radiological findings, a differential diagnosis of plexiform neurofibroma was given, although sarcomatous transformation could not have been entirely excluded from imaging alone. The tumor was subsequently biopsied, and the histopathological analysis confirmed the diagnosis of neurofibroma. This case highlights the importance and diagnostic dilemmas in the presence of this tumor at atypical locations in this disease spectrum.

## Introduction

Neurofibromatosis Type 1 (NF-1), also known as von Recklinghausen’s disease, is a complex multisystemic, autosomal dominant phakomatosis. It is classically distinguished by cutaneous and non-cutaneous manifestations [1]. Plexiform neurofibroma, a subtype of neurofibroma, is believed to be virtually characteristic of NF-1. Plexiform neurofibromas usually occur in the head and neck, pelvis, and extremities [2]. Abdominal manifestations of plexiform neurofibromatosis are unusual and are rarely encountered in patients with NF-1. In this report, we present a rare case of histologically proven neurofibromatosis of the liver, hepatic hilum, retroperitoneum, and mesentery. This case highlights the radiological features, the importance, and diagnostic dilemmas of the presence of this tumor at atypical locations.

## Case presentation

A 52-year-old male, who had been diagnosed with NF-1 in his childhood, was referred to our hospital with an acute history of abdominal pain and vomiting for two days. He had a known medical history of hepatitis C; however, all other laboratory test results obtained at admission, including platelets, clotting profile, liver function tests and alpha-fetoprotein levels, were generally within normal limits (Table 1).

**Table 1:** Laboratory Investigations PT: prothrombin time; INR: international normalized ratio; GGT: gamma-glutamyl transferase; SGPT: serum glutamic pyruvic transaminase; ALP: alkaline phosphatase; AST: aspartate aminotransferase; PCR: polymerase chain reaction.

Parameter	Value	Reference Range
PT	13.0 seconds	9.1 - 13.1
INR	1.2	0.9 - 1.3
Platelets	267 x 10^9^	150 - 400 x 10^9^
Total Bilirubin	0.7 mg/dL	0.1 - 1.2
Direct Bilirubin	0.2 mg/dL	0 - 0.2
Indirect Bilirubin	0.5 mg/dL	0.1 - 0.8
GGT	46 IU/L	Males: < 55
SGPT	43 IU/L	Males: < 45
ALP	43 IU/L	45 - 129
AST	36 IU/L	Males: < 35
Serum Alpha Fetoprotein	5.4 IU/mL	< 6.7
Hepatitis C Qualitative PCR	Reactive	

Initial ultrasound examination raised the possibility of an infiltrative liver lesion. Sonographically, the lesion was of mixed echogenicity, predominantly iso to hypoechoic, with an alteration of the texture of the involved hepatic parenchyma. Subsequently, abdominal computed tomography (CT) was performed to rule out hepatocellular carcinoma based on the history of hepatitis C.

**Figure 1:**
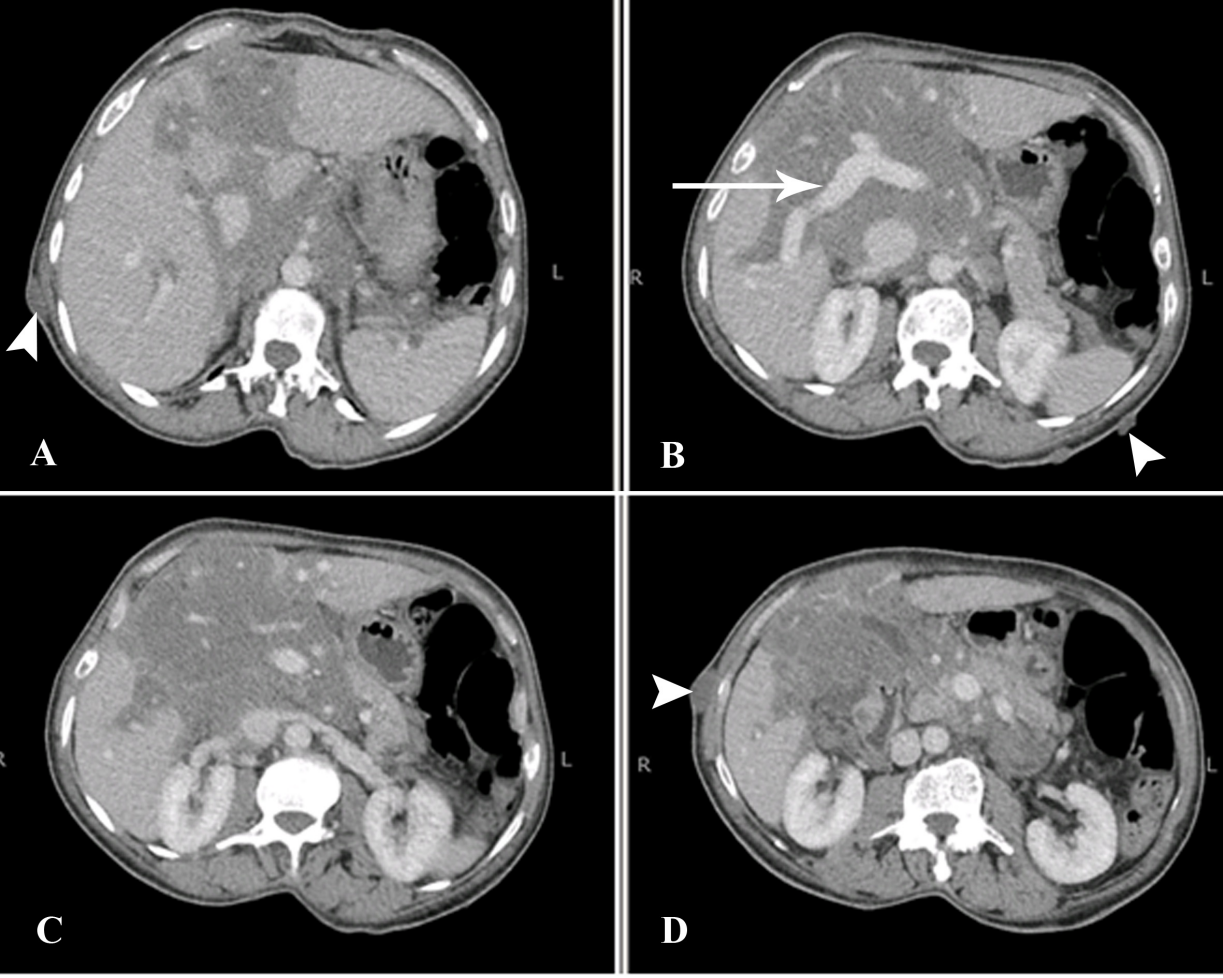
CT Abdomen Axial Sections Multilobulated infiltrative, low-attenuating, minimally enhancing mass at the porta hepatis with intrahepatic and periportal distribution, extending along the retroperitoneum, and encasing the major vessels. The portal vein is normally enhancing (arrow). Multiple cutaneous nodules are also seen, consistent with neurofibromas (arrowheads). CT - computed tomography.

The CT scan revealed a large multilobulated, low-attenuating, minimally enhancing mass involving the liver and porta hepatis. The lesion measured approximately 13.5 x 10.8 cm in size at the porta hepatis (Figure 1).

**Figure 2:**
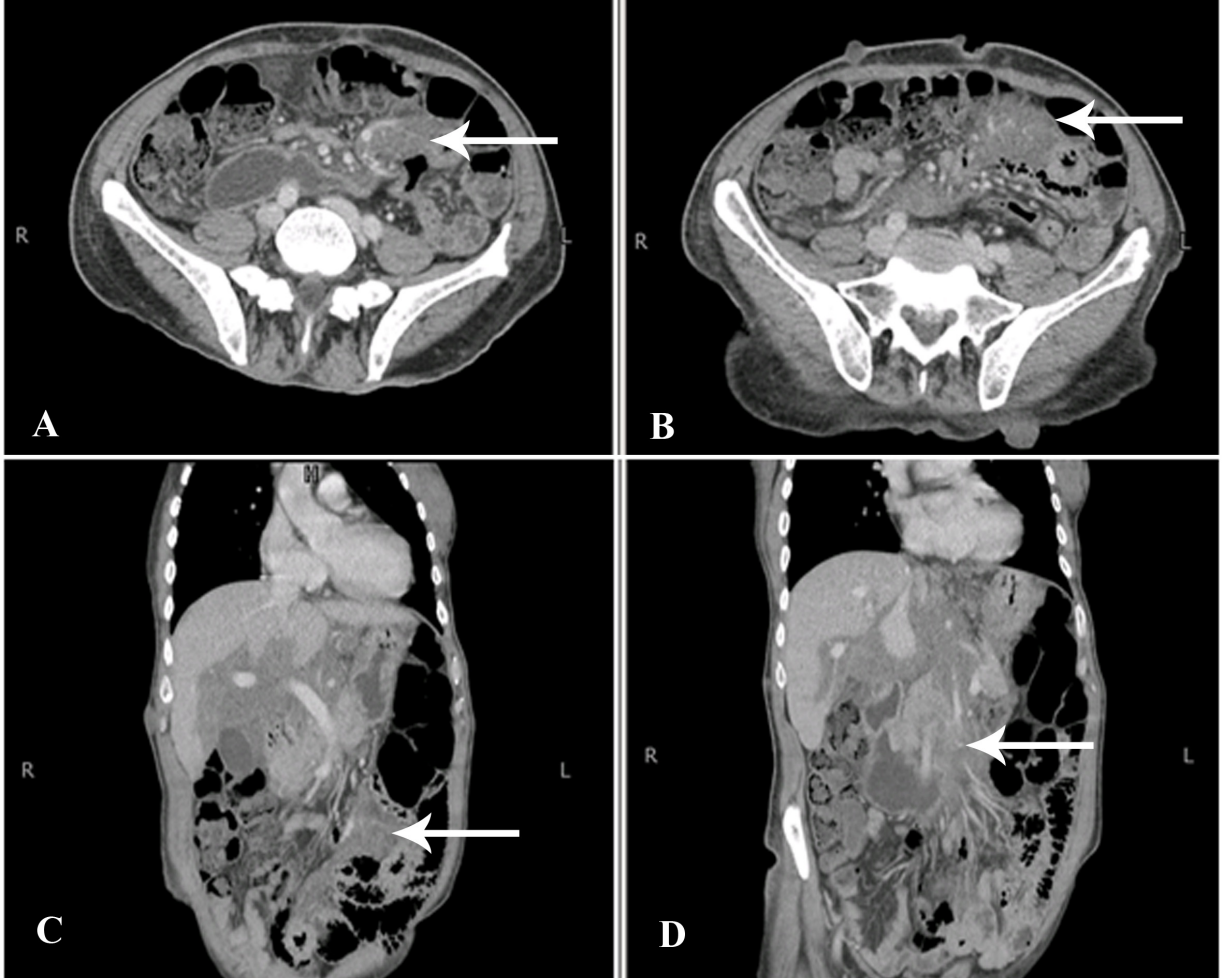
CT Abdomen Axial and Coronal Sections Extensive abdominal involvement of the mass was noted with extension along the superior mesenteric vessels into the mesentery (arrows). CT - computed tomography.

There was no significant mass effect or intra- or extrahepatic biliary dilatation. The lesion was, however, infiltrating along portal tracts via Glisson’s sheath into the liver and was extending into the retroperitoneum through the hepatoduodenal ligament. It was encasing the major vascular structures, such as the portal vein, inferior vena cava, abdominal aorta, and its branches; it was also seen to extend inferiorly into the mesentery along the branches of superior mesenteric vessels (Figure 2).

Keeping in mind the patient’s known history of von Recklinghausen’s disease, a differential diagnosis of plexiform neurofibroma was given and hepatocellular carcinoma was ruled out based on lesion features, enhancement pattern, and lack of other signs of cirrhosis. However, because the lesion was so large and infiltrative, based on imaging alone, the possibility of malignant sarcomatous transformation of the neurofibroma could not have been entirely excluded. The tumor was subsequently biopsied under ultrasound guidance.

**Figure 3:**
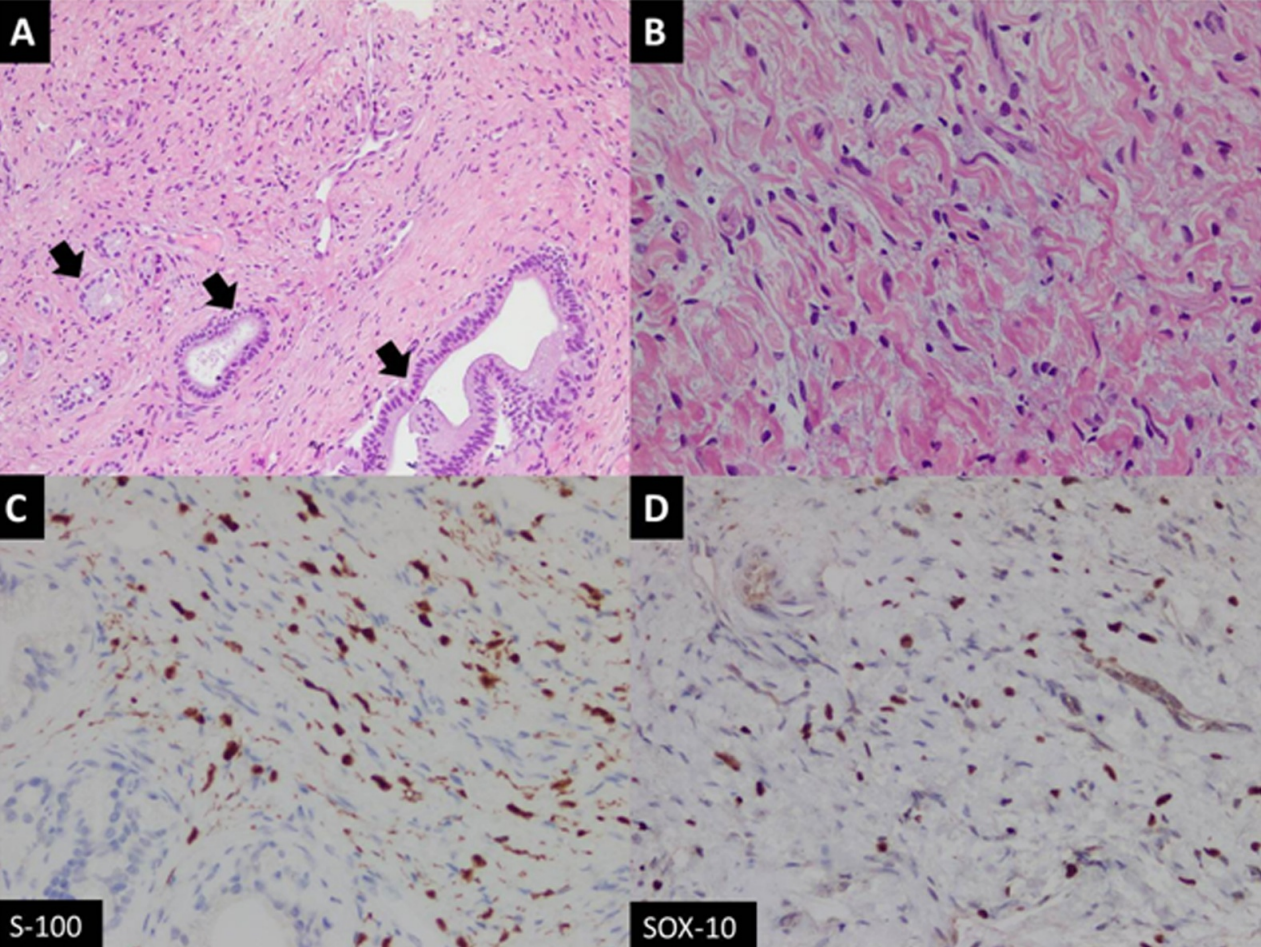
Histology Slides A) Fascicles of spindle-shaped cells with entrapped native bile ducts (arrows) (hematoxylin-eosin (H&E) stain, 100x magnification); (B) High-power view of bland spindle cells loosely arranged against myxoid background (H&E stain, 400x magnification); (C) Tumor cells showing positive nuclear expression for S-100; (D) Sox10 immunohistochemistry stains.

Examination of the histological sections showed a benign spindle cell lesion exhibiting loose fascicles of bland spindle-shaped cells against a collagenous background stroma. These spindle-shaped cells had elongated nuclei with variably visible nucleoli. There was no evidence of increased mitosis, nuclear atypia, or necrosis (Figure 3).

The features favored neurofibroma without sarcomatous elements, hence confirming the diagnosis. The patient was not offered surgical treatment due to the infiltrative nature of the tumor; however, he will be closely followed with surveillance imaging.

## Discussion

Neurofibromatosis Type 1 (NF-1), also known as von Recklinghausen’s disease, is a complex multisystemic, autosomal dominant phakomatosis caused by mutations of the NF-1 gene located on chromosome 17q11.2. Its estimated prevalence is reported to be one in 3,500 [1]. This disease is characterized by multiple non-cutaneous and cutaneous manifestations, some of which include café-au-lait spots, neurofibromas, bony abnormalities, optic nerve gliomas, and Lisch nodules.

Plexiform neurofibroma, which is a variant of neurofibroma, is believed to be virtually diagnostic of NF-1. These are benign nerve sheath tumors which tend to grow along the length of a nerve involving the fascicles and branches [2]. The lesions may occur superficially on the skin or may be located internally. Internally, they are usually found in the head and neck, extremities, or pelvis. Abdominopelvic involvement in NF1 is primarily extraperitoneal, commonly along the sciatic nerve or paraspinal spaces [3]. Involvement of the liver, porta hepatis, retroperitoneum, and mesentery is rare. It is reported that approximately 15 cases of neurofibromatosis in these atypical locations have been documented globally, most of which were found to occur in the pediatric age group [4]. Plexiform neurofibromas, although benign, have a significantly higher potential of transforming into malignant peripheral nerve sheath tumors (MPNSTs). In NF-1 patients, the incidence of developing MPNSTs is thought to be approximately 10% [5].

Radiological features of plexiform neurofibromas on magnetic resonance imaging (MRI) include nodular T2-hyperintense lesions with central hypointense areas giving rise to a target sign. The lesions are usually low in signal on T1-weighted images. On CT, they appear as multilobulated, fusiform, low-attenuation masses, usually along the path of a major nerve and its branches [6]. A normal vessel distribution pattern through the mass is highly pathognomonic and was also seen in imaging of our patient. In previous reports, authors have reported the periportal sheath-like distribution of liver plexiform neurofibromas indicating their proliferation along the intrahepatic nerve fibers at the porta hepatis. Others have mentioned the periportal collar sign (low attenuation around the periportal spaces) as a helpful distinguishing imaging feature [7-8].

According to Malagari et al., adjunctive imaging techniques, like CT portography, show both the perivascular involvement and the preservation of normal vessel distribution throughout the lesions. Angiography may also be used to exclude true splanchnic vessel involvement, stenoses, aneurysmal formation, and non-aneurysmal ectasias that are associated with spindle cell infiltration. Although not typically hypervascular, these tumors may illustrate faint vascularity, seen only on arteriography. Asymmetry in the size or attenuation of these lesions, inhomogenous enhancement, or increased vascularity on angiography may suggest malignant transformation [9]. However, sarcomatous degeneration of plexiform neurofibromas is challenging to diagnose by imaging alone, which is why the complete exclusion of MPNSTs often warrants a biopsy.

Internal plexiform neurofibromas are slow growing, usually asymptomatic, and are mostly incidentally discovered on imaging. The current mainstay of treatment is surgery; however, due to the significant infiltration of surrounding tissues and nerves, complete excision always poses a surgical challenge. Differential diagnosis of these lesions includes other infiltrative hypoattenuating tumors, such as lymphomas, sarcomas, atypical cystadenocarcinomas, and mixed epithelial and mesenchymal tumors. Other primary liver neoplasms that are associated with NF-1, such as schwannomas, angiosarcomas, hepatomas, neurofibrosarcomas, and malignant schwannomas, should also be kept in mind [9-10].

## Conclusions

In conclusion, we have reported an unusual case of neurofibromatosis of the liver, porta hepatis, retroperitoneum, and mesentery in an adult patient with von Recklinghausen’s disease. These tumors, although rarely encountered at such locations, have distinctive imaging features with normal vessel distribution patterns. Due to their diffuse and infiltrative nature, complete surgical resection is generally not possible in most cases and, therefore, histopathological correlation and regular surveillance are often needed to exclude malignant transformation.

## References

[1] Boyd KP, Korf BR, Theos A (2009). Neurofibromatosis type 1. J Am Acad Dermatol.

[2] Korf BR (1999). Plexiform neurofibromas. Am J Med Genet.

[3] Hoshimoto S, Morise Z, Takeura C (2009). Plexiform neurofibroma in the hepatic hilum associated with neurofibromatosis type 1: a case report. Rare Tumors.

[4] Fujisawa T, Takata M, Ouchi S (2011). Intra-abdominal plexiform neurofibromatosis including periportal, mesentery, and gastrointestinal tract involvement in neurofibromatosis type 1: case report and review of the literature. Clin J Gastroenterol.

[5] Evans DG, Baser ME, McGaughran J (2002). Malignant peripheral nerve sheath tumours in neurofibromatosis 1. J Med Genet.

[6] Lin J, Martel W (2001). Cross-sectional imaging of peripheral nerve sheath tumors: characteristic signs on CT, MR imaging, and sonography. AJR Am J Roentgenol.

[7] Gossios KJ, Guy RL (1993). Case report: imaging of widespread plexiform neurofibromatosis. Clin Radiol.

[8] Rodriguez E, Pombo F, Rodriguez I, Vázquez Iglesias JL, Galed I (1993). Diffuse intrahepatic periportal plexiform neurofibroma. Eur J Radiol.

[9] Malagari K, Drakopoulos S, Brountzos E, Sissopulos A, Efthimidadou A, Hadjiyiannakis E, Kelekis DA (2001). Plexiform neurofibroma of the liver: findings on MR imaging, angiography, and CT portography. AJR Am J Roentgenol.

[10] Ghalib R, Howard T, Lowell J (1995). Plexiform neurofibromatosis of the liver: case report and review of the literature. Hepatology.

